# Evaluation of Antioxidant Activity, Polyphenolic Compounds, Amino Acids and Mineral Elements of Representative Genotypes of *Lonicera edulis*

**DOI:** 10.3390/molecules19056504

**Published:** 2014-05-21

**Authors:** Jiri Sochor, Tunde Jurikova, Miroslav Pohanka, Helena Skutkova, Mojmir Baron, Lenka Tomaskova, Stefan Balla, Borivoj Klejdus, Robert Pokluda, Jiri Mlcek, Zuzana Trojakova, Jan Saloun

**Affiliations:** 1 Department of Viticulture and Enology, Faculty of Horticulture, Mendel University, Valticka 337, CZ-691 44 Lednice, Czech Republic; E-Mails: mojmirbaron@seznam.cz (M.B.); tomaskova.l.9@gmail.com (L.T.); 2 Department of Chemistry and Biochemistry, Faculty of Agronomy, Mendel University, Zemedelska 1, CZ-613 00 Brno, Czech Republic; E-Mail: borivoj.klejdus@mendelu.cz; 3 Institute for Teacher Training, Faculty of Central European Studies, Constantine the Philosopher University, Drazovska 4, SK-949 74 Nitra, Slovak Republic; E-Mails: tjurikova@ukf.sk; (T.J.); sballa@ukf.sk (S.B.); 4 Faculty of Military Health Sciences, University of Defence, Trebesska 1575, CZ-500 01 Hradec Kralove, Czech Republic; E-Mail: miroslav.pohanka@gmail.com; 5 Karel English College, Sujanovo namesti 356/1, 60200 Brno, Czech Republic; 6 Department of Biomedical Engineering, Faculty of Electrical Engineering and Communication, Brno University of Technology, Kolejni 4, CZ-612 00 Brno, Czech Republic; E-Mail: skutkova@feec.vutbr.cz; 7 Department of Vegetable Science and Floriculture, Faculty of Horticulture, Mendel University, Lednice, Valtická 337, CZ-691 44 Lednice, Czech Republic; E-Mail: pokluda@mendelu.cz; 8 Department of Food Analysis and Chemistry, Faculty of Technology, Tomas Bata University, Namesti T.G.Masaryka 275, 762 72 Zlin, Czech Republic; E-Mail: mLcek@ft.utb.cz; 9 Department of Garden and Landscape Architecture, Faculty of Horticulture, Mendel University, Valticka 337, CZ-691 44 Lednice, Czech Republic; E-Mail: xtrojak2@node.mendelu.cz; 10 Department of Applied Pharmacy, Faculty of Pharmacy, University of Veterinary and Pharmaceutical Sciences, Palackeho 1, CZ-612 42 Brno, Czech Republic; E-Mail: jan_saloun@post.cz

**Keywords:** *Loniceraedulis*, mineral compounds, phenolic compounds, antioxidant activity, amino acids, bioinformatics analysis

## Abstract

The aim of this study was to evaluate the bioactive substances in 19 berry cultivars of edible honeysuckle (*Lonicera* *edulis*). A statistical evaluation was used to determine the relationship between the content of selected bioactive substances and individual cultivars. Regarding mineral elements, the content of sodium was measured using potentiometry and spectrophotometry. The content of selected polyphenolic compounds with high antioxidant activity was determined by a HPLC–UV/ED method. The total amount of polyphenols was determined by the Folin-Ciocalteu method. The antioxidant activity was determined using five methods (DPPH, FRAP, ABTS, FR and DMPD) that differ in their principles. The content of 13 amino acids was determined by ion-exchange chromatography. The experimental results obtained for the different cultivars were evaluated and compared by statistical and bioinformatic methods. A unique feature of this study lies in the exhaustive analysis of the chosen parameters (amino acids, mineral elements, polyphenolic compounds and antioxidant activity) during one growing season.

## 1. Introduction

The edible honeysuckle (*Lonicera* *edulis*) is a lesser known fruit species native of the territory of Russia [1], especially the Kamchatka Peninsula and Siberia. The berries are valued for their high content of ascorbic acid [2,3], mineral elements, especially potassium [4] and polyphenolic compounds [5]. The related blue berried honeysuckle (*Lonicera caerulea*) contains the highest level of potassium compared with *Ribes idaeus*, *R. nigrum* and *R. nigrum* x *R. dikuscha*. Moreover, the polyphenolic content in *L. caerulea* is significantly higher than the content in *Ribes idaeus* and *Ribes nigrum* [6]. The major classes of phenolic compounds in the blue berried honeysuckle are flavonols (quercetin, rutin, quercitrin) [7], flavanols (proanthocyanidins, catechins) and anthocyanins [2,8]. Futhermore, the edible honeysuckle berries represent the richest source of chlorogenic acid, quercetin, and rutin in comparison with other lesser known fruit species such as *Amelanchier alnifolia*, *Prunus tomentosa* [3]*.* The berries have high antioxidant capacity due to a high content of antioxidants (ascorbic acid, polyphenols). Chaovanalikit *et al.* [9] measured the total antioxidant capacity in 11 fruit samples of different subspecies of *L. caerulea* as oxygen radical absorbing capacity (ORAC), which gave values of 18 to 104 μmol Trolox equivalent per g of fresh weight and ferric reducing antioxidant power (FRAP), with values of 37 to 113 μmol Trolox equivalent per g of fresh weight. According to these authors [9] the antioxidant capacity was most correlated with total phenolic contents (r = 0.97). In addition, a study by Rop *et al.* [7] using the DPPH (2,2-diphenyl-1-picrylhydrazyl) test determined a high antioxidant activity in particular cultivars of *L. kamtschatica* (6.59–10.17 g of ascorbic acid equivalent/kg of fresh weight - FW) introduced into the conditions of the Czech Republic. Blue honeysuckle provided comparable ORAC (18.4–103.7 mmol of Trolox equivalent/kg) with berries of several other genera like blackberries (13.0–146.0 mmol of Trolox equivalent/kg) and black currants (17.0–116.0 mmol of Trolox equivalent/kg) [9]. The potential of the edible honeysuckle berries to exhibit antimicrobial and antiviral [10], antifungal [11] and anticancer properties [12] are all related to their high polyphenolic content [9].

This paper reports a study of the mineral elements, polyphenolic compounds, and amino acid content in 17 cultivars of edible honeysuckle selected in Russia and two Slovak cultivars–Amur and Altaj. The cultivars were planted under the same environmental conditions. There is little information concerning the amino acid content in edible honeysuckle berries. Furthermore, analyses of the antioxidant capacity of berries were provided by two simultaneous tests–FRAP, ORAC [13] or four tests (antioxidant activity against DPPH, ABTS, H and O_2_) [14]. Our study offers an evaluation of the antioxidant activity of *Lonicera* berries using five tests—DPPH, FRAP, ABTS, FR and DMPD. A new approach by cluster analyses made it possible to sort the different cultivars according to their suitability for utilization in food processing and for pharmaceutical purposes.

## 2. Results and Discussion

The content of six mineral compounds, seven polyphenolic compounds, total polyphenolic content, antioxidant activity and 13 amino acids were detected in 19 particular cultivars of edible honeysuckle. The results and differences between them were evaluated by cluster analyses.

### 2.1. Determination of Mineral Compounds

First the contents of potassium, nitrogen, sodium, calcium, magnesium, and phosphorus in edible honeysuckle berries were determined. Wild berries and edible honeysuckle cultivars represent a similar source of K, Mg and Ca [15]. According to Plekhanova [1], potassium predominates in the berries of edible honeysuckle, reaching levels up to 300–500 mg/kg FW. All assayed potassium values were higher (3.13 g/kg for Nimfa cultivar – 4.56 g/kg FW for Gerda cultivar). In the results of Jurikova and Matuškovič [2], the berries of edible honeysuckle reached maximum levels of potassium (12.2 g/kg; 12.8–13.0 g/kg FW), which are lower in comparison with the results of our experiments. The levels of magnesium (79 mg/kg Sinnaja Ptica – 163 mg/kg Tomichka), calcium (312 mg/kg Gerda – 489 mg/kg Kamchadalka, Nimfa and Amur) and sodium (10.2 mg/kg Tomichka – 18.7 mg/kg Altaj FW) in the berries of edible honeysuckles were higher in comparison with studies carried out in Slovakia (710 mg/kg, 1,070 mg/kg; 80 mg/kg FW) [2] and Russia in the case of magnesium and calcium (890–1,220 mg/kg; 1,650–2,400 mg/kg FW). On the other hand, Russian cultivars possessed higher values of sodium (300–530 mg/kg FW) [1]. Jurikova *et al.*, [3] analysed the same cultivar-Amur ‒ and reported higher levels of the observed mineral elements (N – 4,027, P – 585, K – 3,477, Ca – 527, Mg- 140 and Na-16 mg/kg dry weight - DW). On the contrary, Altaj contained a higher amount of nitrogen (4,260 mg/kg DW), phosphorus (552 mg/kg DW), potassium (3,620 mg/kg DW), calcium (443 mg/kg DW) and sodium (18.7 mg/kg DW) in our experiments. Except for Amfora (5,520 mg/kg DW) the cultivars assayed in [9] displayed higher values of phosphorus (386 mg/kg Morena – 612 mg/kg Kamchadalka FW). All mentioned differences can be caused by the differences in the cultivation years examined, as observed in the research of Jurikova and Matuškovič [2] on *L.* *kamtchatica* and *L. edulis* berries.

**Figure 1:**
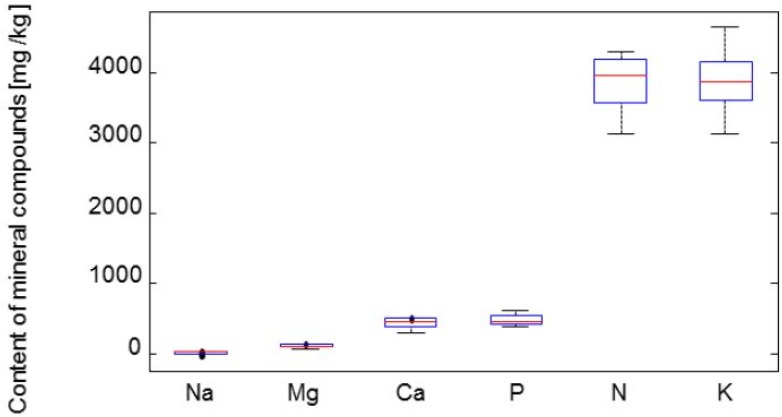
The content of mineral elements expressed by box plot graphs. Na–sodium, Mg–magnesium, Ca–calcium, P–phosphorus, N–nitrogen, K–potassium.

**Figure 2:**
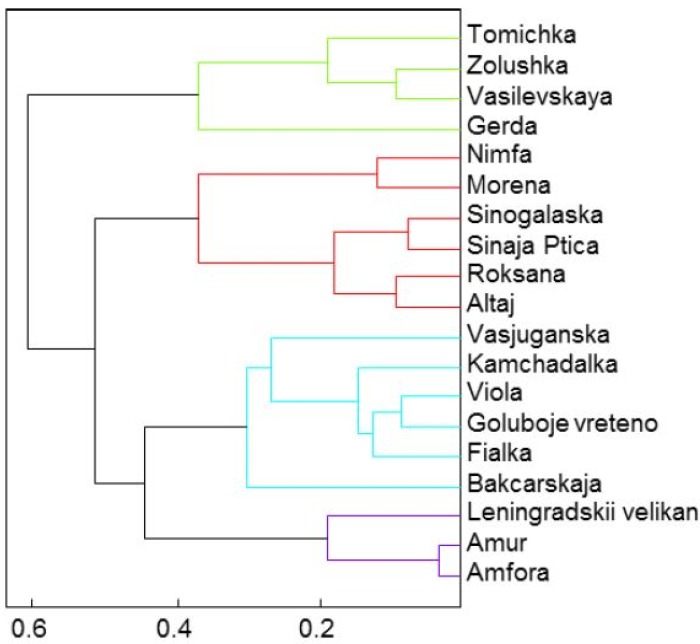
The cluster analysis of mineral elements represented by four principal clusters.

Figure 1 illustrates the distribution of all analysed mineral elements by their concentration. On the basis of the results achieved we can rank the mineral components in the following order: K > N > P > Ca > Mg > Na, in accord with the results of Jurikova and Matuškovič [2] who assayed the mineral matter content in *L. kamtschatica* and *L. edulis* berries. The average values of mineral elements were the following: K – 3,890 mg/kg, N ‒ 3,820 mg/kg, P ‒ 486 mg/kg, Ca ‒ 442 mg/kg, Mg ‒ 117 mg/kg, Na ‒ 14.0 mg/kg (all units referred to FW). A dendrogram (Figure 2) was generated by cluster analysis in which the cultivars of the edible honeysuckle were divided into four representative groups on the basis of the similarity of their mineral element contents.

Thus, according to the values of mineral matter content the studied cultivars can be distributed into the following groups by cluster analysis:

*Group 1*: Tomichka, Zolushka, Vasivskaya and Gerda
*Group 2*: Nimfa, Morena, Sinoglaska, Sinaja Ptica, Roksana and Altaj
*Group 3*: Vasjuganska, Kamchadalka, Viola, Goluboje vreteno and Fialka
*Group 4*: Leningradskii velikan, Amur and Amfora.

The cultivars in the first group are grouped based on their highest content of potassium, the second group can be characterized by the highest content of nitrogen, the third phosphorus and finally the fourth group had the lowest values of nitrogen.

### 2.2. Determination of Total Polyphenols Content

Edible honeysuckle berries represent a rich source of ascorbic acid and polyphenolic compounds, especially anthocyanins, proanthocyanidins and flavonoids [16]. The results of the total content of polyphenolic compounds are summarized in Table 1.

**Table 1:** The values of the total polyphenolic content in observed cultivars of honeysuckle. Results are given in mg/kg equivalent of gallic acid (FW).

Cultivars	Total Polyphenols	Cultivars	Total Polyphenols
Altaj	5,542 ± 82	Nimfa	6,259 ± 54
Amfora	5,632 ± 94	Roksana	7,896 ± 103
Amur	7,326 ± 111	Sinaja Ptica	7,598 ± 117
Bakcarskaja	6,859 ± 108	Sinogalaska	7,789 ± 124
Fialka	6,596 ± 71	Vasilevskaya	6,985 ± 132
Gerda	8,236 ± 105	Vasjuganska	7,536 ± 135
Goluboje vreteno	8,659 ± 109	Viola	7,159 ± 84
Kamchadalka	7,798 ± 88	Tomichka	8,259 ± 129
Leningradskij velikan	6,235 ± 81	Zolushka	8,569 ± 115
Morena	6,235 ± 77		

The reported values of total phenolic compounds for blue honeysuckle cultivated under Czech Republic conditions ranged from 5,750 to 9,030 mg/GAE/kg FW [7], which were quite similar to our results (from 5,540 for Altaj up to 8,660 mg/kg FW in Goluboje vreteno). Skupien *et al.* [17] reported a lower value of total polyphenols (1,660 mg/kg FW) in the cultivar Czerna up to 2,840 mg/kg FW and 3,190 mg/kg FW in the cultivar Zielona. Similarly, Ochmian *et al.* [18] revealed the polyphenol content in the early ripening cultivar Wojtek to be 1,490 mg/kg FW. According to Rupasinghe *et al*. [19], the total polyphenolic content in Haskap Borealis reached 6,990 mg QE/kg FW that represented a higher value in comparison with blueberries, blackberries and strawberries. On the basis of 51 different Russian genotypes of edible honeysuckle native to Russia, Streltsyna *et al.* [20] reported the total content of polyphenolic compounds to be very variable and the total amount of polyphenols was higher than our results, reaching up 7,820–18,900 mg/kg FW. Among the examined samples of small berries native to Western Canada, it was found out that berries of edible honeysuckle fruits contained the highest amount of polyphenolic compounds–11,100 mg of gallic acid equivalent per kg ‒ among all examined small berries [21]. In comparison with other berry crops, for example, strawberry and blackberry, lower values of total polyphenols (2,380 mg GAE/kg FW) [22] were determined.

### 2.3. Determination of Selected Polyphenolic Compounds

**Figure 3:**
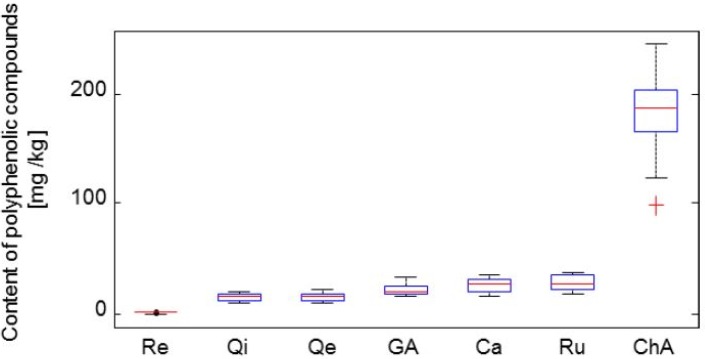
Box plot graphs demonstrating the rate of dispersions of individual methods for original measured data. Re–resveratrol, Qi–quercitrin, Qe–quercetin, GA–gallic acid, Ca–catalposide, Ru–rutin, ChA–chlorogenic acid.

Papers on chromatography of alcohol extract of the edible honeysuckle often focus on the presence of 5–12 phenolic compounds in relation to species. Analyses of these fractions and their localization on the chromatogram make it possible to identify them as hydroxycinnamic acids, flavonols and flavones [23]. In the first part of the experiment, we focused our effort on measurement of the content of compounds of interest–gallic acid, catalposide, rutin, resveratrol, quercitrin, chlorogenic acid and quercetin. The results of the determined polyphenolic compounds are given in Figure 3.

In Figure 3, antioxidants are set up in box plot graphs on the basis of their observed distribution in fruit. The average values of the observed antioxidants were: Re ‒ 1.8 mg/kg, Qi ‒ 16 mg/kg, Qe ‒ 17 mg/kg, GA ‒ 22 mg/kg, Ca ‒ 27 mg/kg, Ru ‒ 29 mg/kg and ChA ‒ 182 mg/kg FW. The total content (free and bound) of phenolic acids in *L. edulis* berries ranges from 2.85 ± 0.14 to 5.42 ± 0.23 g/kg DW [24] with the proportion of chlorogenic acid in berries being 0.48% [16]. It is evident that in all examined samples chlorogenic acid represented the major antioxidant. In our study, the content of chlorogenic acid reached from 98.0 mg/kg FW (Amfora) up to 236 mg/kg FW (Zolushka, that are higher values than in an experiment done by Orinčak *et al.* (270–600 mg/kg) who examined Klčovs clones under conditions of Slovakia [25]. Skupien *et al.* [17] reported 234.8 mg/kg FW that is quite a similar value to that of Tomicka. When comparing the results of Gazdik *et al.* [5], the analyzed content of polyphenolic compounds in *L.* *edulis* berries was almost the same. Chlorogenic acid had a prevailing position among phenolic acids in *L. kamtschatica* berries in the studies of Deneika *et al.* [26]. The authors claimed that chlorogenic acid is the most abundant phenolic acid in Nature [27]. On the other hand, Zadernowski *et al.* [24] did not identify chlorogenic acid in their study of the course of the substance’s instability. According to studies by Ochmian *et al.* [18], in the case of two cultivars of *L. kamtschatica*, chlorogenic acid represented 70%–81% of the polyphenolic compounds. On the other hand, Oszmianski and Wojdylo [28] did not find similar results, since chlorogenic and neochlorogenic acids accounted for only 7.5% of the total amount of polyphenols [28]. In our study, chlorogenic acid in Zolushka cultivar represented 27.5% of the total phenolic compounds. The mentioned differences can be caused by the different cultivation conditions and genotypes analyzed or more precisely the cultivars. Derivatives of hydroxybenzoic acid are present in berries in the form of its esters with the most diverse structures of gallic acid and salicylic acid [4]. From this group of antioxidants, gallic acid predominated in all examined samples (160 mg/kg FW in Fialka; 340 mg/kg FW in Zolushka). Our reported content of gallic acid is lower in comparison with the results of Zadernowski *et al.* [24] who studied blue berried honeysuckle cultivars in Poland and also [29]. They determined the content of gallic acid in blue berried honeysuckle to be 44.3 ± 2.6 mg/kg for DM. Beside this, they studied *L. edulis* Turcz. ex. Freyn having a content of gallic acid 600 mg/kg DM and cultivar Sinoglaska with a gallic acid content of 60 mg/100 g DM [30].

Rutin was the most abundant antioxidant from the group of polyphenols with a flavonoid scaffold (19.6 mg/100 g FW Vasiljevskaja; 37.9 mg/100 g FW Zolushka), followed by quercitrin (1122 mg/kg FW Fialka; 243 mg/kg FW Sinnaja Ptica). A quite similar content of quercitrin (23 mg/kg FW) was noted by Gazdik *et al.* [5] in *L. edulis*, but under the same cultivation conditions Rop *et al.* [7] determined higher values of quercitrin in cultivar Sinoglaska (22.2 mg/kg FW). The concentration of quercitrin in our samples reached higher values in comparison with ligonberry, branberry, chokeberry, rowanberry and cowberry. The highest amount of rutin (480 mg/kg) was determined in samples of *L. boczkarnikowae* by Plekhanova–Streltsyna [1] but in Klčovs clones of *L. kamtschatica* lower values (130–410 mg/kg) [4] were measured. Gazdik *et al.* [5] examined the content of rutin in *L. edulis* Turcz. ex. Freyn cultivated under the same conditions and found 240 mg/kg FW. A quite similar concentration of rutin was observed in samples of cultivar Sinoglaska (240 mg/kg FW) [31].

In contrast with our studies, in two Polish cultivars, Wojtek and Brazowa, Ochmian *et al.* [32] determined quercetin-3-glucoside and qurcetin-3-rutinoside as the main flavonoids. In general, quercetin is the predominant flavonol in fruit. Manach *et al.* [27] reported lower levels than 15–30 mg/kg FW, which is in accord with our results. On the other hand, lower quercetin values were noticed in Klčovs clones of *L. kamtschatica* (13–70 mg/kg) [25]. Similar quercetin values were determined by Häkkinen *et al.* [33] in samples of blueberry (105–160 mg/kg FW) and cranberries (73–172 mg/kg FW). In cultivars Goluboe vreteno and Zolushka, a significant amount of *trans*-resveratrol (23.6 mg/kg; 23.5 mg/kg FW) was measured. Differences in the analyzed polyphenol levels can be caused by the different locality of cultivation. On the other hand, the content of polyphenols is also influenced by year, maturity stage of the tested fruit and storage conditions of the harvested fruit [34]. The composition of honeysuckle extract depends on plant genotype [35].

**Figure 4:**
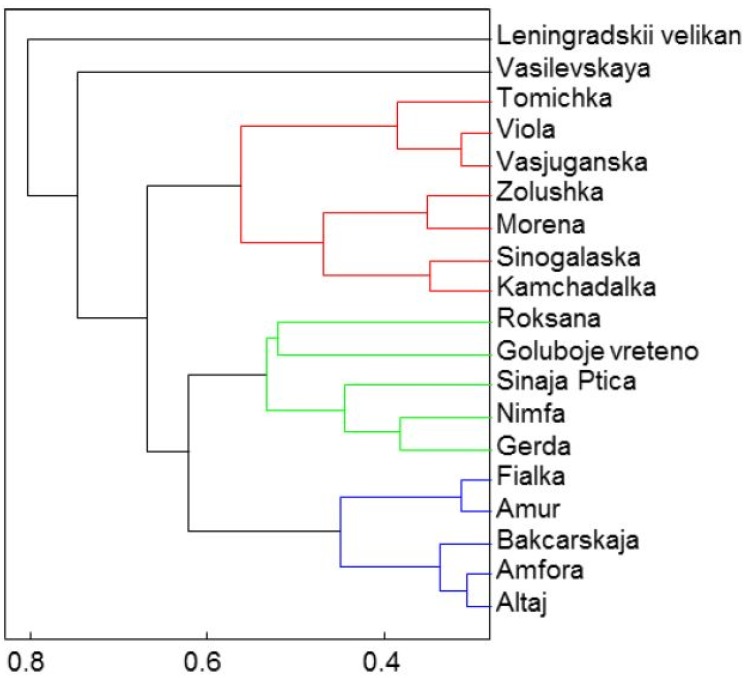
The cluster analysis of polyphenolic compounds represented by five principal clusters.

Results on the basis of polyphenolic compounds are distributed into five basic groups by cluster analysis as we can see at Figure 4:

*Group 1*: Leningradskij velikan
*Group 2*: Vasilevskaya
*Group 3*: Tomichka, Viola, Vasjuganska, Zolushka, Morena, Sinoglaska, Kamchadalka
*Group 4*: Roksana, Goluboje vreteno, Sinaja Ptica, Nimfa, Gerda
*Group 5*: Fialka, Amur, Bakcarskaja, Amfora, Altaj.

As regards the representatives of the first group, the typical trait is that they have the lowest content of chlorogenic acid. On the other hand, the second group can be distinguished by the highest content of chlorogenic and gallic acids.

### 2.4. Determination of Antioxidant Activity

The determination of antioxidant activity, concurrently with the assessment of the main representative bioactive components in berries–polyphenolic compounds‒is one of the ways of expressing the biological value of the edible honeysuckle berries [31]. Numerous assays have been described to measure various free radical damage products or antioxidant status and the plethora of available techniques attest to the fact that no single ideal method is available [36,37,38]. In addition, levels of antioxidants in food do not necessarily reflect their antioxidant activity [39,40,41,42].

The concept of a single test that reflects antioxidant activity is an attractive one [43,44]. Low antioxidant activity could be indicative of oxidative stress or increased susceptibility to oxidative damage. For interpretation of the measurement results it is essential that the user fully understand the relative contributions of the individual antioxidants. Therefore, a better approach is to use a range of measurements of individual antioxidants coupled with antioxidant activity. Nowadays, antioxidant activity measurements find wide applications in research, food industry and drug discovery [45].

Since plant foods contain many different classes and types of antioxidants, knowledge of their antioxidant activity, which is the cumulative capacity of food components to scavenge free radicals, would be useful for epidemiologic purposes [37]. In accordance with investigated protective effect of several plant foods, antioxidant activity is thought to be one of the basic standards of biological foodstuff value.

**Figure 5:**
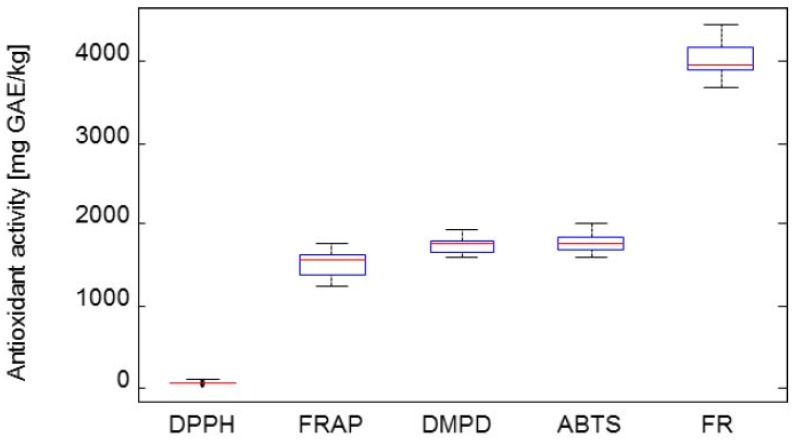
Box plot graphs of antioxidant activity. The results are given in equivalents of gallic acid.

The determination of the antioxidant activity in blueberries by spectrophotometric methods has already been done in many studies [3]. There are no studies devoted to the evaluation of antioxidant activity of *L. berries* by five different methods (DPPH test–chemical compound 2,2-diphenyl-1-picrylhydrazyl; ABTS method chemical compound 2,2'-azino-bis(3-ethylbenzothiazoline-6-sulphonic acid; FRAP method–Ferric ion reducing antioxidant power; DMPD method–chemical compound dimethylfenylfenylendiamin and FR method–Free Radical method). These methods are all based on quenching or trapping free radicals [46,47]. Results are expressed as equivalents of gallic acid in mg/kg. The antioxidant activity values are shown in Figure 5. Different values of antioxidant activity between these methods are caused by the different study approaches used for examining the antioxidant activity in fruits.

Figure 5 shows box plot graphs with the distribution of antioxidant activity data of berries determined by five different methods. The average values of antioxidant activity with the different tests were the following: DPPH–66 mg/kg, FRAP–1,505 mg/kg, ABTS–1,777 mg/kg, FR ‒ 4,037 mg/kg, DMPD ‒ 1,748 mg/kg (all unites refer to FW). The lowest antioxidant activity value determined by the DPPH, FRAP and ABTS assays was noted in the Altaj cultivar (50.3 mg/kg GAE/kg; 1,232 mg/kg GAE/kg; 1,585 mg GAE/kg) and the highest in the Zolushka one (86.5 mg/kg GAE/kg, 1,781 mg/kg GAE/kg; 2,007 mg/kg GAE/kg). The antioxidant activity value of Zolushka cultivar is similar to the antioxidant efficiency of *Lonicera caerulea* measured by Raudsepp *et al.* [48]. Paulovicsova *et al.* [49] reported lower antioxidant activity values determined by the DPPH test (30‒33 mg/kg GAE/kg) in samples of Klčovs clones of *Lonicera kamtschatica*. On the other hand, Gazdik *et al.* [5] found the highest antioxidant activity value by the DPPH test. They reported 89.9 mg/kg GAE/kg, that represented the highest value among Chinese hawthorn and Saskatoon berry.

The lowest antioxidant activity value observed by the FR method was reported in Leningradskij velikan: 3.70 g/kg GAE/kg and 1.59 g/kg GAE/kg in Morena was the lowest by the DMPD method. The highest antioxidant activity value measured by FR was 4.46 g/kg GAE/kg and by DMPD 1.93 g/kg GAE/kg in Zolushka cultivar. It could therefore be expected that the Zolushka one could be considered as the cultivar with the best potential for development in added food products and natural health products utilized for preventing chronic diseases. In this way, Zolushka would have the same utilization as Haskap Borealis [19,29]. Moreover, Zolushka possessed the highest value of total phenolic content, gallic and chlorogenic acids, catalposide and quercitrin among all tested cultivars.

**Figure 6:**
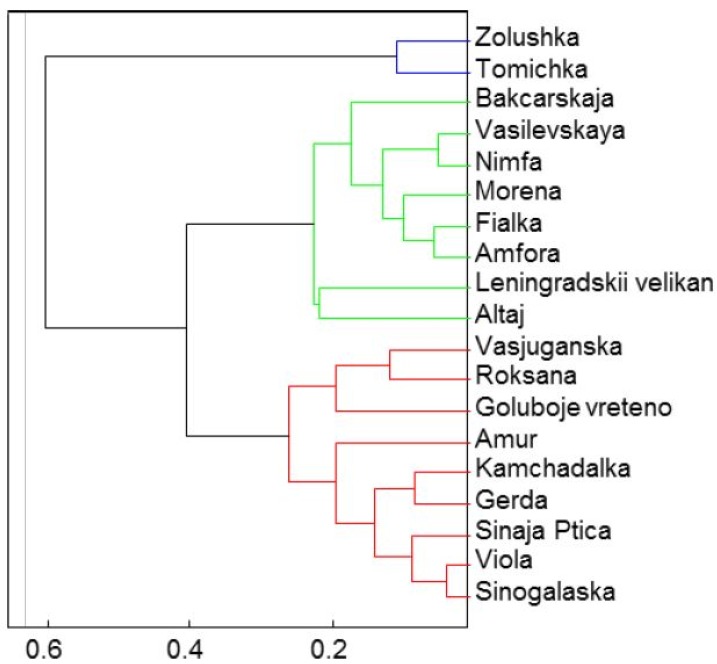
The cluster analysis of antioxidant activity represented by three principal clusters.

All methods display a high positive correlation ranging from r^2^ = 0.786 to r^2^ = 0.998. The highest correlation coefficients were obtained between the ABTS and FRAP methods (r^2^ = 0.998). High correlations also existed between the DPPH and DMPD methods (r^2^ = 0.895), DPPH and FRAP methods (r^2^ = 0.897), and DMPD and FRAP methods (r^2^ = 0.886) that is in agreement with the results of Rupasinghe *et al.* [19]. Results on the antioxidant activity are distributed in three basic groups by cluster analysis, as we can see in Figure 6. Zolushka and Tomichka represented the highest values by the FRAP, ABTS, FR and DPMD assays; on the other hand, the second group had the lowest values of antioxidant activity as determined by the DPPH, FRAP, ABTS, FR and DPMD methods:

*Group 1*: Zolushka, Tomichka
*Group 2*: Bakcarskaja, Vasilevskaya, Nimfa, Morena, Fialka, Amfora, Leningradskii velikan, Altaj
*Group3*: Vasjuganska, Roksana, Goluboje vreteno, Amur, Kamchadalka, Gerda, Sinaja Ptica, Viola, Sinoglaska.

In the next step, we determined the relationship between the antioxidant activity of fruit and the total polyphenol content. A highly positive correlation (from r^2^ = 0.756 up to r^2^ = 0.936) between all methods for the determination of antioxidant activity and the total phenolic content indicated that phenolic compounds represented some of the main components responsible for the reducing ability of these fruits. The findings are in accord with the studies by Rupasinghe *et al.* [19]. The highest correlation coefficients were obtained between the total phenolic content and FRAP methods (r^2^ = 0.936).

### 2.5. Determination of Amino Acids

Most often analyses of free amino acid contents in berries have been performed by liquid chromatography [48]. Surprisingly, very little work has been done regarding the amino acid content of fruits, and most such studies were conducted in wine grapes [50]. Red currants contain a higher level of arginine than black currants. The lack of differences in the contents of proline among bilberries is characteristic. Cultivated bilberries contain a higher level of arginine, glutamic acid and alanine than wild growing ones [51]. According to studies done by Rupasinghe [19], crude protein in berries is 4.6% in Haskap Indigo Gem and up to 8.41% in Haskap Tundra. The values are lower in comparison with strawberries and raspberries. To the best of our knowledge, there are no reports on honeyberries concerning amino acid content, and only Plekhanova [23] reported that the dominant amino acid was asparagine, followed by glutamine, leucine and alanine. Our studies confirmed the predominant position of asparagine, followed by serine.

**Figure 7:**
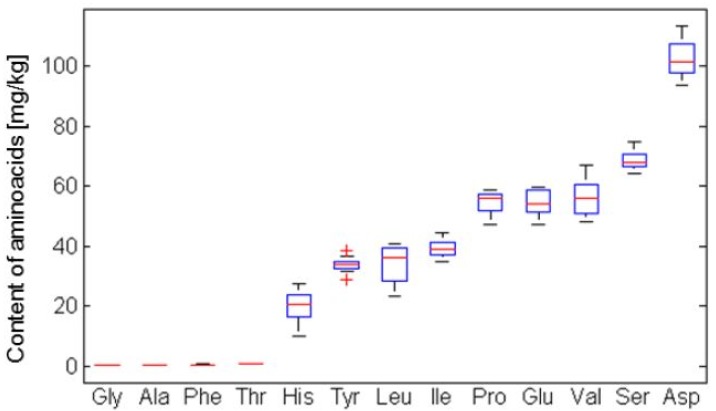
Box plot graphs in the distribution of determined amino acids. (Gly–Glycine, Ala–Alanine, Phe–Phenylalanine, Thr–Theorine, His–Histidine, Tyr–Tyrosine, Leu–Leucine, Ile–Illeucine, Glu–Glutamic acid, Pro–Proline, Val–Valine, Ser–Serine, Asp–Aspartic acid). The results are depicted in mg/kg.

Figure 7 shows the box plot graphs with the distribution of amino acids on the basis of their content. Amino acids were set up from the predominantly occurring (aspartic acid) to the less abundant (alanine). The average values for individual amino acids were the following: Gly – 0.301 mg/L, Ala – 0.430 mg/L, Phe – 0.465 mg/L, Thr – 0.678 mg/L, His – 19.7 mg/L, Tyr – 33.8 mg/L, Leu – 34.3 mg/L, Ile – 39.3 mg/L, Glu – 54.5 mg/L, Pro – 54.5 mg/L, Val – 55.9 mg/L, Ser – 68.9 mg/L, Asp – 102.8 mg/L (all units were in the FW).

**Figure 8:**
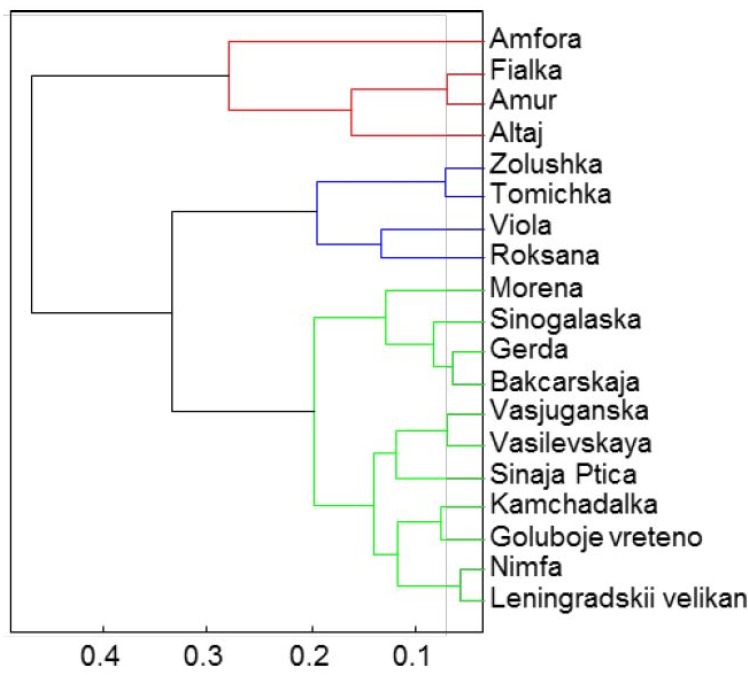
The cluster analysis of mineral compounds represented by three principal clusters.

According to the cluster analysis results (Figure 8) the amino acids are distributed in three basic groups (Figure 8) by cluster analysis, where the individual clusters represented by Leningradskij velikan and Vasilevskaja displayed the highest values of alanine and asparagine. The representatives of the first group can be distinguished by the highest content of histidine, the second by glutamine:

*Group 1*: Amfora, Fialka, Amur and Altaj
*Group 2*: Zolushka, Tomichka, Viola and Roksana
*Group 3*: Morena, Sinoglaska, Gerda, Bakcarskaja, Vasjuganska, Vasilevskaya, Sinaja Ptica, Kamchadalka, Goluboje vreteno, Nimfa, Leningradskij velikan.

## 3. Experimental

### 3.1. Biological Material

All samples have the same origin. The trial plots of Mendel University in Brno, Czech Republic are situated in Žabčice, approximately 20 km from Brno (Czech Republic). The altitude of the field is 184 m, average temperature 9 °C (15.6 °C in the vegetation period), precipitation 553 mm (356 mm in the vegetation period). According to soil genesis, this area belongs to the gley alluvie brown soil group created in the Holocene as carbonate gley sediments with great accumulation of organic matters. In terms of ploughland texture, it is clayey. Fruits were harvested in May 2013 at the consumption maturity stage and stored at −18 °C until needed for an analysis not later than two months after harvesting. Ten g were used as a representative sample for the analyses.

### 3.2. Sample Preparation

Representative samples (10 g) were taken and transferred to a mortar. Liquid nitrogen was added and the samples were homogenised in 99% (w/w) methanol (10 mL). The homogenate was transferred to a disposable tube and was ground under the same conditions for 30 min. Sonication was the next step and centrifugation of the samples at 16,400 rotations per minute for 30 min using an Eppendorf 5804R centrifuge (Eppendorf, Hamburg, Germany) followed. Supernatants were filtered through a 0.45 µm pore size membrane filter (Metachem, Torrance, CA, USA) and 500 µL of each filtrate were diluted with 500 µL of 99% (w/w) methanol.

For the amino acid determination, dried and pulverized sample (0.025 g) was mixed with HCl (6 M, 0.5 mL). The prepared sample was transferred into a vial and placed in a termostable holder which was placed in the microwave system and subsequently mineralized in a Multiwave3000 microwave apparatus (Anton-Paar GmbH, Graz, Austria). Microwave conditions were power 100 W, ramp 10 min, hold time 140 min, cooling time 10 min.

### 3.3. Chemicals and PH Measurements

Standards of target phenolic compounds used for HPLC-ED analysis: gallic acid and 4*‑*aminobenzoic acid (Sigma Aldrich Corp., St. Louis, MO, USA), salicylic acid, chlorogenic acid and flavonoids: chrysin (Sigma Aldrich) rutin trihydrate and quercitrin dihydrate (Roth GmbH, Karlstruhe, Germany), quercetin, diosmin (Merck, Darmstadt, Germany), catalposide, resveratrol (Sigma Aldrich- Fluka Co. St. Louis. MO, USA). Liquid nitrogen was purchased from Messer technogas s.r.o, (Prag, Czech Republic). Methanol (>99.9%; *v*/*v*), formic acid, acetic acid and 96% ethanol for HPLC were from Dr. Kulich Pharma (Hradec Kralove, Czech Republic). 2,2-Diphenyl-1-picrylhydrazyl (DPPH), and acetonitrile for HPLC were from Sigma Aldrich, as were the other purchased chemicals (analytical purity unless noted otherwise).

Stock standard solutions of the thiols (1 mg/mL) were prepared with ACS water (Sigma-Aldrich) and stored in dark at −20 °C. The working standard solutions were prepared daily by dilution of the stock solutions. All solutions were filtered through 0.45 μm nylon filter discs (Millipore, Billerica, MA, USA) prior to HPLC analysis. The pH value was measured using a probe WTW inoLab Level 3 instrument with Level 3 (WTW, Weilheim, Germany).

### 3.4. Determination of Minerals Content

Spectrophotometric measurements of antioxidant activity were carried out using a BS-400 automated chemical analyser (Mindray, Shenzhen, China). It was composed of a cuvette space tempered to 37 ± 1 °C, reagent space with a carousel for reagents (tempered to 4 ± 1 °C), sample space with a carousel for the preparation of samples and an optical detector. Transfer of samples and reagents was provided by robotic arm equipped with a dosing needle (error of dosage not exceeding ±5% of volume). Cuvette contents were mixed by an automatic mixer including a stirrer immediately after addition of reagents or samples.

Into plastic cuvettes, an aliquot (200 µL) of a kit for the determination of nitrogen and calcium, supplied by the Groner Company (Ootmarsum, Netherlands) was pipetted. Subsequently, the measured samples (20 µL) were added. Absorbance was measured for 10 min. at λ = 678 nm for determination of magnesium and at λ = 630 nm for the determination of phosphorus. For element content estimation, values of absorbance of the reagent and absorbance of the sample after 10 min of incubation were used. Values were subtracted and results recalculated in accordance with a calibration curve for the given element. Sodium and potassium ions were determined electrochemically using ion selective electrodes, which are integral parts of the BS-400 apparatus. Concentrations of these ions were measured by indirect potentiometry.

### 3.5. HPLC Profile of Selected Antioxidants

For the determination of the HPLC profiles of the individual cultivars, high performance liquid chromatography (HPLC) with electrochemical and UV-VIS detection was used. The system consisted of two Model 582 ESA chromatographic pumps (ESA Inc., Chelmsford, MA, USA) with a working range from 0.001 to 9.999 mL/min. and a Zorbax SB C18 (150 × 4.6; size of particles 5 µm, Agilent Technologies, Santa Clara, CA, USA) reverse phase chromatographic column. For UV detection, a Model 528 ESA UV detector was used. A twelve-channel CoulArray detector (ESA) was used for electrochemical detection. Samples were injected automatically by an autosampler (Model 542, ESA), which includes a thermostatic space for a column. The assay conditions can be found in Zitka *et al.* [52].

### 3.6. Determination of Total Content of Polyphenols

The Folin-Ciocalteu method, based on the reduction of a phosphotungsten-phosphomolybdate complex by phenols to blue reaction products was used for the determination of phenolic compounds. The sample (0.5 mL) was pipetted into a cuvette and diluted with ACS water (1.5 mL). Subsequently, Folin-Ciocalteu reagent (50 µL) was added and the solution was incubated at 22 °C for 30 min. The absorbance was measured using a dual-beam SPECORD 210 spectrophotometer (Carl Zeiss, Jena, Germany) at a wavelength λ = 760 nm against a blank (all chemicals without a sample or gallic acid) according to reference [53]. The absorbance was measured in triplicate. The method was calibrated using the well-known phenolic compound gallic acid and results were expressed as equivalents of gallic acid in mg/100 g.

### 3.7. Determination of Antioxidant Activity

Spectrophotometric measurements of antioxidant activity were carried out using the BS-400 automated chemical analyser (Mindray, Shenzhencity, China). Transfer of samples and reagents was provided by a robotic arm equipped with a dosing needle (error of dosage not exceeding ±5% of volume). Cuvette contents were mixed by an automatic mixer including a stirrer immediately after addition of reagents or samples.

#### 3.7.1. Determination of Antioxidant Activity by the ABTS Test

The procedure for the determination was taken from a publication by Sochor *et al.* [54]. A 150 µL volume of reagent. Seven mM 2,2'-azinobis-3-ethylbenzothiazoline-6-sulfonic acid (ABTS^•^) and 4.95 mM potassium peroxodisulphate was mixed with 3 µL of the sample. Absorbance was measured at 660 nm for 10 min. 

#### 3.7.2. Determination of Antioxidant Activity by the FRAP Method

The procedure for this determination was taken from a paper by Sochor *et al.* [55]. A 150 μL volume of reagent was injected into a plastic cuvette with subsequent addition of a 3 μL sample. Absorbance was measured at 605 nm for 10 min. 

#### 3.7.3. Determination of Antioxidant Activity by the Free Radicals Method

Procedure for the determination was taken from a publication by Pohanka *et al.* [47]. A 150 μL volume of reagent was injected into a plastic cuvette with subsequent addition of a 6 μL sample. Absorbance was measured at 450 nm for 10 min. 

#### 3.7.4. Determination of Antioxidant Activity by the DPPH Test

This procedure for the determination was taken from publications by Sochor *et al.* [54]. A 150 µL volume of reagent (0.095 mM 2,2-diphenyl-1-picrylhydrazyl ‒ DPPH^•^) was incubated with 15 µL of the sample. Absorbance was measured at 505 nm for 10 min. 

#### 3.7.5. Determination of Antioxidant Activity by the DMPD Method

The determination procedure was taken from a publication by Sochor *et al.* [54]. A 160 μL volume of reagent (200 mM DMPD, 0.05 M FeCl_3_, 0.1 M acetate buffer pH 5.25) was injected into a plastic cuvette with subsequent addition of 4 μL of sample. Absorbance was measured at 505 nm for 10 min.

### 3.8. Determination of Amino Acids

For the determination of amino acids an ion-exchange liquid chromatography (Model AAA-400, Ingos, Prag, Czech Republic) with post column derivatization with ninhydrin and a UV-VIS detector was used. A glass column with inner diameter of 3.7 mm and 350 mm in length was filled manually with a strong cation exchanger in sodium cycle LG ANB (Ingos) with approximately 12 µm particles and 8% porosity. The column was tempered within the range from 35 to 95 °C. The elution of the amino acids of interest was carried out with the column temperature set to 74 °C. A double channel UV-VIS detector with inner cell of volume 5 µL was set to two wavelengths, 440 and 570 nm. A solution of ninhydrin (Ingos) was prepared in 75% *v*/*v* methyl cellosolve (Ingos) and in 2% *v*/*v* 4 M acetic buffer (pH 5.5). Tin chloride (SnCl_2_) was used as a reducing agent. The prepared solution of ninhydrin was stored under an inert atmosphere (N_2_) in the dark at 4 °C. The flow rate was 0.25 mL/min. and the reactor temperature was 120 °C.

### 3.9. Mathematical and Statistical Analysis

Mathematical and statistical analyses of experimental data were carried out with MATLAB^®^, Version 7.9.0.529 (R2009b, MathWorks Inc., Natick, MA, USA). For the CA calculation procedure we used the *pdist*function to calculate the distance between every pair of objects (honeysuckle) in a data set. We compute the Euclidean distance between pairs of objects in m-by-n data matrix X. Rows of X correspond to observations, and columns correspond to variables. D is a row vector of length m(m–1)/2, corresponding to pairs of observations in X. D is dissimilarity matrix for the next clustering. The *pdist* function calculates the Euclidean distance between objects, but we can specify one of several other options. We used the standardized Euclidean, so each coordinate difference between rows in X is scaled by dividing by the corresponding element of the standard deviation. 

## 4. Conclusions

This paper offers a comprehensive screening of edible honeysuckle berries in terms of their biologically active substances. For the evaluation we used a wide range of nutrient parameters–amino acids, mineral elements, polyphenolic compounds and antioxidant activity of fruit collected during one growing season. All analyses were performed immediately after the collection of samples. This makes degradation of the observed compounds impossible. Our study also provides a new approach in the mathematical evaluation of the results by cluster analysis. Cluster analyses make it possible to determine the possible relationships between assayed cultivars on the basis of the observed parameters. The results of our study proved that the analyzed edible honeysuckle berries represent prospective sources of health-supporting biologically active substances–mineral elements, amino acids and polyphenolic compounds‒displaying high antioxidant activity. Because of their durability under poor conditions, these plants can be recommended for cultivation under different climatic conditions and they can be a valuable source of nutraceutical preparations. Zolushka cultivar displayed the highest fruit antioxidant activity related to a highest content of evaluated polyphenolic compounds.
